# Bureaucracy stifles medical research in Britain: a tale of three trials

**DOI:** 10.1186/1471-2288-12-122

**Published:** 2012-08-16

**Authors:** Helen Snooks, Hayley Hutchings, Anne Seagrove, Sarah Stewart-Brown, John Williams, Ian Russell

**Affiliations:** 1 College of Medicine, Swansea University, Singleton Park, Swansea, SA2 8PP, UK; 2 Warwick Medical School, Warwick University, Coventry, CV4 7AL, UK

## Abstract

**Background:**

Recent developments aiming to standardise and streamline processes of gaining the necessary approvals to carry out research in the National Health Service (NHS) in the United Kingdom (UK), have resulted in lengthy and costly delays. The national UK governmental Department of Health’s Research Governance Framework (RGF) for Health and Social Care requires that appropriate checks be conducted before research involving human participants, their organs, tissues or data can commence in the NHS. As a result, medical research has been subjected to increased regulation and governance, with the requirement for approvals from numerous regulatory and monitoring bodies. In addition, the processes and outcomes of the attribution of costs in NHS research have caused additional difficulties for researchers. The purpose of this paper is to illustrate, through three trial case studies, the difficulties encountered during the set-up and recruitment phases of these trials, related to gaining the necessary ethical and governance approvals and applying for NHS costs to undertake and deliver the research.

**Methods:**

Empirical evidence about delays and difficulties related to regulation and governance of medical research was gathered during the period 2009–2010 from three UK randomised controlled trials with sites in England, Wales and Scotland (1. SAFER 2- an emergency care based trial of a protocol for paramedics to refer patients directly to community based falls services; 2. COnStRUCT- a trial of two drugs for acute ulcerative colitis; and 3. Family Links - a trial of a public health intervention, a 10 week community based parenting programme). Findings and recommendations were reported in response to a call for evidence from The Academy of Medical Sciences regarding difficulties encountered in conducting medical research arising from R&D governance and regulation, to inform national policy.

**Results:**

Difficulties and delays in navigating and gaining the appropriate approvals and NHS costs required to undertake the research were encountered in all three trials, at various points in the bureaucratic processes of ethical and research and information governance approvals. Conduct of each of the three trials was delayed by at least 12 months, with costs increasing by 30 – 40%.

**Conclusions:**

Whilst the three trials encountered a variety of challenges, there were common issues. The processes for gaining approvals were overly complex and differed between sites and UK countries; guidance about processes was unclear; and information regarding how to define and claim NHS costs for undertaking the research was inconsistent. The competitive advantage of a publicly funded, open access health system for undertaking health services research and clinical trials within the UK has been outweighed in recent years by stifling bureaucratic structures and processes for governance of research. The recommendations of the Academy of Medical Sciences are welcomed, and the effects of their implementation are awaited with interest.

**Trial Registration numbers:**

SAFER 2: ISRCTN 60481756; COnStRUCT: ISRCTN22663589; Family Links: ISRCTN 13929732

## Background

McKinsey [1] and Cooksey [2] advocated and achieved substantial investment in the infrastructure for medical research within the United Kingdom (UK). They applauded the excellent opportunities for world-class medical research within the National Health Service (NHS), and recognised widespread failure to exploit these opportunities through cumbersome regulations and processes. The UK Clinical Research Collaboration (UKCRC) aimed to establish a national infrastructure for clinical research, with the goal of “creating a clinical research environment that will improve national health, increase national wealth, and enrich world knowledge”. A key element of the UKCRC is a national network of research networks – topic specific (e.g. Mental Health Research Network) and generic (Comprehensive Local Research Networks – CLRNs), to encourage participation in high quality clinical studies, managed through the National Institute for Health Research (NIHR) “portfolio” and to provide a coordinated and efficient infrastructure of research personnel and facilities to support patient recruitment to these studies [3].

However difficulties were still encountered following the efforts to streamline and invigorate these processes [4,5].

Research within the UK health services has seen a shift in recent years from being a largely unregulated activity, carried out independently of external controls to becoming a formalised, regulated and institutionalised process [6]. This has centred largely around the publication in 2005 of the Department of Health’s Research Governance Framework (RGF) for Health and Social Care that requires, in line with European Union and wider international standards, that appropriate checks be carried out before research involving human participants, their organs, tissues or data can commence [7]. Researchers within the UK have to seek approvals from Research Ethics Committees (RECs) and local Research and Development (R&D) committees prior to starting their research in the NHS.

**Figure 1:**
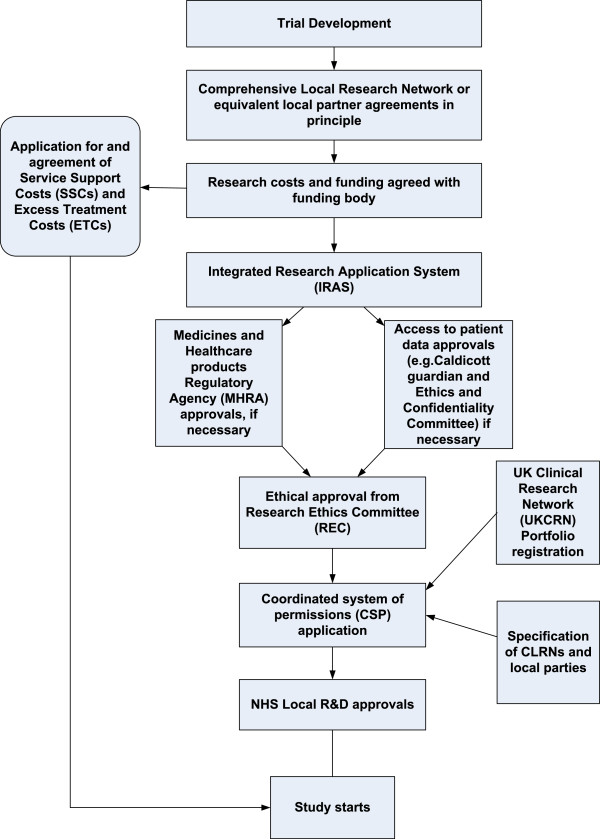
Application process for gaining approvals in the UK prior to beginning a trial.

**Figure 2:**
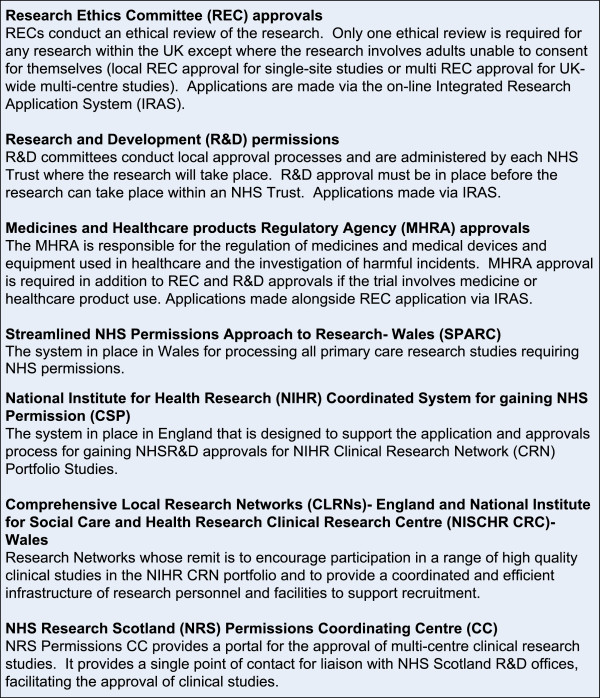
Details of application processes required for the 3 trials.

Following trial set-up and agreement of funding, local agreement is sought in principle with the Comprehensive Local Research Networks (CLRN) or equivalent in order to steer the process of gaining local governance approvals (see Figure 1). The governance approvals process then formally begins with the submission of a nationally standardised on-line application form, currently administered through the Integrated Research Application System (IRAS) which was launched in January 2008 (see Figure 2). This begins with the process of gaining global Research Ethics Committee (REC) approvals. If required, applications are also made at this point, through IRAS, to the Medicines and Healthcare products Regulatory Agency (MHRA) or other appropriate committees (for example if access to patient data is required). Following REC approvals, local level NHS Research and Development (R&D) approvals are sought. ‘Coordinated’ systems for gaining R&D approvals have been implemented to facilitate this process in England, and to some extent in Scotland and Wales. In England the NIHR Coordinated System for gaining NHS Permission (CSP) is a system designed to support the application and approvals process for NHS R&D approval for publicly funded peer-reviewed research. Topic-specific networks or CLRNs steer this process, with a lead assigned to each case, dependent on the location of the study Chief Investigator. In Wales, at the time of this study, individual Trust R&D applications had to be made with the support of The National Institute for Social Care and Health Research Clinical Research Centre (NISCHR CRC) for portfolio studies in secondary care, with a separate process for studies in primary care (Streamlined NHS Permissions Approach to Research- Wales (SPARC). For multi-centre research in Scotland, the NHS Research Scotland (NRS) Permissions Coordinating Centre (CC) coordinates the process of obtaining permissions from Scottish Health Boards.

**Figure 3:**
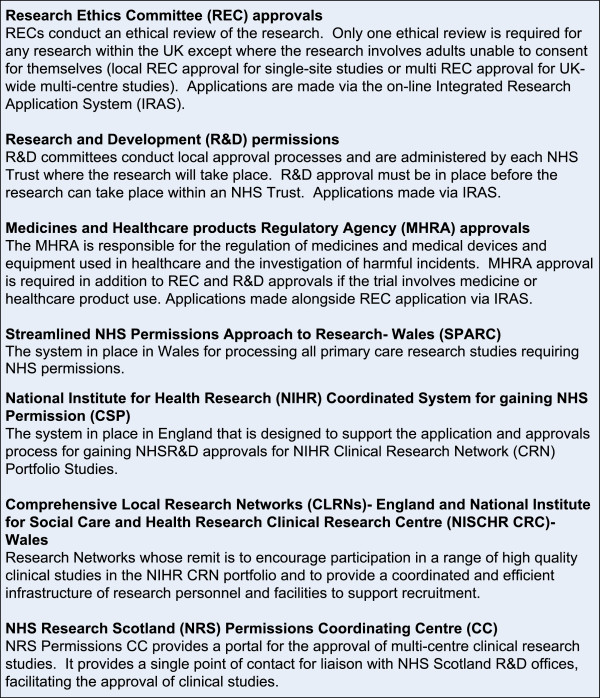
Timeless for the SAFER 2, COnStRUCT, and Family Links Trials, showing delays attribute to R&D governance and regulation processes.

In many instances NHS partners incur costs related to administration of the research processes of studies (service support costs) and/or to increase the throughput of patients or the level of care given to them (excess treatment costs). These costs cannot be included in the application to NIHR research funding bodies but are recoverable, in principle, through applications to Primary Care Trusts (PCTs) and CLRNs in England and their equivalents in Wales and Scotland.

Recent publications in both the medical and mainstream press have further highlighted difficulties and barriers to undertaking medical research [8-10]. In response to these issues, the Academy of Medical Sciences recently called for evidence regarding difficulties encountered in conducting medical research arising from R&D governance and regulation [11,12].

We supplied evidence in response to this call, and summarise our recent experience related to the set up and delivery of three NIHR portfolio trials here.

## Methods

We gathered empirical evidence regarding the governance and regulatory processes (including ethical approval, research and information governance, MHRA approvals and applications for support funding in the form of excess treatment and service support costs) from the set-up and recruitment phases of three randomised controlled trials, each of which was registered as a portfolio study, during the period 2009–2011. The three trials were being carried out in sites across England, Wales and Scotland: 1. SAFER 2- an emergency care based trial of a protocol for paramedics to refer patients directly to community based falls services; 2. COnStRUCT- a trial of two drugs for acute ulcerative colitis; and 3. Family Links - a trial of a public health intervention, a ten week community based parenting programme (see Table 1).

**Table 1:** Overview of the three NIHR portfolio studies included in this paper

	**SAFER 2**	**COnStRUCT**	**Family Links**
**Research Design**	Cluster randomised controlled trial	Individually randomised controlled trial	Individually randomised controlled trial
**Health Technology (Intervention) being assessed**	Treatment protocol and care pathway	Comparison of two drugs for the treatment of acute severe Ulcerative Colitis	10 week group based parenting programme (2 hours per week)
**Population**	Older people (aged >= 65) who have suffered a fall and for whom a call has been made to the emergency ambulance service	Age 18 and over suffering with acute severe Ulcerative Colitis	Parents with children aged 2 - 4
**Sites**	South Wales, Nottingham, North East London	Greater than 40 secondary care sites throughout the UK	4 centres in South Wales
**Sample size**	6336	480	288
**Funding body**	NIHR Health Technology Assessment Programme	NIHR Health Technology Assessment Programme	Consortium of Local Authorities with Welsh Assembly Government
**Initial funding awarded**	£1.2 million	£1.69 million	£350,000
**Initial timescale**	2009 - 2012	2008-2012	2008 - 2011

We gathered data from research protocols, project management documents and correspondence with project funders and regulatory bodies, and summarised our findings into common themes. We mapped the time taken to carry out all the regulatory processes and obtain the necessary governance approvals in order to set-up the three trials against the original project milestone charts. We consulted with the trial managers and principal investigators for each of the three trials and gained original documentation and letters which specified the submission and approval dates for all the governance and regulatory processes. Where further clarification was necessary, additional information was sought from minutes of steering or project team meetings. Where delays were experienced, the resource implications of the protracted application processes were extracted from extension applications to the project funders.

Findings and recommendations from the three trials were reported in to the call for evidence from The Academy of Medical Sciences regarding difficulties encountered in conducting medical research arising from R&D governance and regulation, to inform national policy.

### Results

#### Research Ethics Committee (REC) approvals

We experienced problems gaining a positive ethical decision at the outset of the SAFER 2 trial because, in this cluster randomised controlled trial, we had proposed an opt-out consent process to access medical records in order to follow up patient outcomes. This mirrored another trial in the same population (older people for whom a 999 call had been made for a fall), that was underway and that had been approved by the same REC. Consent is acknowledged to be problematic [13,14] in research in the emergency care setting.

The absence of clear guidelines in this field resulted in diversion of the application to the National Information Governance Board for health and social care (NIGB). The REC and the NIGB offered conflicting opinions regarding what consent processes should be employed in the trial. Illustrating the confused boundaries between regulatory bodies, information governance issues were raised by the REC and ethical issues were raised by the NIGB with no clear path to resolution. This resulted in a substantial use of resources (researcher time, as well as the time of both panels) and delays. A positive ethical opinion for the trial, with substantially amended consent processes, was given one year after our initial application.

The COnStRUCT and Family Links trials experienced no major problems or delays in gaining REC approval.

#### Research and development (R&D) approvals

Once ethical approval had been gained for the SAFER 2 trial, we sought R&D permissions from 26 NHS partners within five CLRNs (or Welsh equivalent) across three participating ambulance service sites. The process of R&D governance was further complicated by the fact that the trial included sites in England and Wales which had different processes for gaining approvals. The CSP was in place in England, which streamlined the process to a certain degree, however we still experienced varying levels of feedback and communication with designated CLRNs as well as different processes at individual site level for approval of studies. In Wales, R&D approval at local level was still dependent on agreement at local committees, many of whom required completion of their own additional R&D application forms and processes, in addition to the generic IRAS form.

The COnStRUCT trial included over 40 trial sites based in England, Wales and Scotland for which research governance approval was required. The process of gaining these R&D approvals was delayed while we sought agreement to transfer from previously completed National Research Ethics Service (NRES) documentation to the IRAS system. This was an important step in allowing the study to progress through the then recently introduced CSP, which was considered vital given the number of sites that would be involved. The CSP process began in May 2009. A further necessary step in the process was approval from the Medicines and Healthcare products Regulatory Agency (MHRA), which took longer than expected due to technical issues and the initial loss of the application by the MHRA. Despite claims that the CSP would streamline administration, in practice it proved very complex. There was no coordinated Welsh process in place at the time. The sheer volume of sites involved led to technical difficulties, with IRAS unable to cope with the high number of potential sites. After 12 months fewer than half of the participating NHS partner sites had given R&D approval for this trial.

The Family Links trial was simpler in terms of geography, as it took place in four sites in Wales, but more complex from the point of view of recruitment. As the intervention was a group-based programme run in school term time, there were only three opportunities to recruit each year. Following REC approval, we sought R&D permissions from each partner NHS site in order to recruit families through community based children’s centres, with support from NHS based research support services. Several local R&D committees highlighted ethical issues, seeming to work in isolation and causing delays to the start of the trial. When the initial recruitment strategy, through the children’s centres, failed to meet projected targets we decided to extend recruitment by involving general practices in the four study areas. We made an application to SPARC to approach general practices. The application coincided with local NHS reconfigurations and the swine flu pandemic which were both cited as being responsible for delays. Approval to contact general practices in two of these areas was finally gained six months after our application. Given the lead time on setting up recruitment through this route, it was already too late to use this approach and the opportunity to recruit from general practice thus passed. Additional efforts and new strategies for boosting recruitment through the children’s centres were successful, but the recruitment period had been lengthened considerably.

#### Attribution and reimbursement of costs

Completion of the process of gaining R&D approvals was further delayed in the SAFER2 trial by the failure of the various parties involved - the CLRNs, participating NHS sites and the research funder - to agree on who should pay for what. This was compounded by the issue and subsequent withdrawal of new national guidance on cost attribution in research (ReSeT) during this process. Protracted discussions between the research team, the lead CLRN and the Department of Health eventually helped to agree cost attributions, but still discussions remained at local level regarding the level of these costs and formal agreement of their reimbursement to NHS partners, necessary before these organisations would complete the sign up processes. The retrieval of these costs also presented a challenge due to the different routes and application processes that were in place in England and Wales.

In the COnStRUCT trial local NHS partner approvals were needed to pay for the trial drugs, which in some sites were delayed, and in others refused altogether. Local discussions took place and business cases were submitted to NHS partner sites. The outcome has been unsuccessful at several partner sites in spite of the fact that one of the drugs used has been recommended by NICE for ‘use in research’ and as an NIHR study, the NHS is obliged to meet the treatment costs.

Attribution of costs was not an issue for the Family Links Trial which took place within a local authority and was not covered by existing agreements between research funders and the NHS.

### Impact on the three trials

The delays to each trial are shown in Figure 3. In the SAFER 2 trial, one minor amendment took in excess of 4 months to gain approval (with some local R&D approvals still outstanding). The amendment was the addition of an attractive front cover to the existing approved questionnaire in order to improve response rates. To be able to use the front sheet we had to gain approval from our trial task and finish group, the ethics committee who originally approved the trial, the sponsor of the trial and each individual R&D committee. The time spent on the tasks associated with gaining approval approximated to around 3 days of work for a senior researcher.

For SAFER 2, delays in gaining approvals and the labyrinthine negotiations regarding attribution of costs delayed the project start by 12 months and increased expenditure by 40%, from £1.2 million to approximately £1.7 million. We were granted additional funding for all the extension costs from the NIHR HTA. The NIHR requested that the trial gain all the necessary approvals prior to further payments being made. However, we pleaded special circumstances on SAFER 2 and the NIHR agreed to release payments.

The protracted process of gaining approvals in addition to unresolved discussions in relation to who should pay for the expensive drugs, compounded by slower than anticipated patient recruitment on COnStRUCT will delay reporting by at least 24 months. A delay of at least one year can be attributed to research governance, albeit staggered across sites. We have recently applied to the NIHR HTA for a 2 year extension and are awaiting the outcome. If granted the result will be an increase in expenditure of 50%, from £1.6 million to approximately £2.4 million.

The funders of the Family Links trial, have extended the trial period twice and increased the budget by more than 75% in order to achieve the target sample size without recruiting from general practices. Results were delayed by one year , with an increase in trial costs from £0.35 million to £0.82 million. All additional extension funding was received from the original funder.

## Discussion

While these three trials encountered different challenges, there were common issues between them. Although for two of the trials, gaining ethical approvals was fairly straightforward (COnStRUCT and Family Links), for SAFER 2 this was problematic despite the fact similar methodology had been employed in a previous trial with the same population of patients. Advice regarding appropriate ethical practice in research in populations at risk is arcane [15,16] and this presents RECs with difficulties when reviewing such studies. The lengthy processes of gaining consent to participate in a trial in the emergency care setting, has recently been shown to directly contribute to increased mortality (The CRASH 2 trial [17]). It was also found that the processes for gaining NHS R&D permissions were complex and differed both between sites and the three UK countries (England, Wales and Scotland) involved. Information about these processes and best practice in responding was also elusive. Gaining R&D permissions was especially difficult without clarity about the financial consequences of participation, with inconsistency regarding how to reclaim and categorise these costs.

Our findings mirror those of other medical researchers. In a pragmatic study where no medical intervention was given, the authors suggest that an additional 150 days should be added to a research study to allow time to gain local R&D approvals [18]. This proved to be an underestimate in each trial case study described in this paper. Researchers of trials of medicinal products have also experienced delays [19]. Even since the apparent ‘streamlining’ of the governance processes, researchers have still experienced difficulties [20]. In collating the evidence resulting from its call regarding delays and difficulties related to regulation and governance of medical research, the Academy of Medical Sciences report highlights the difficulties encountered and points to significant delays in obtaining necessary approvals [21]. The report highlights that in some population groups such as Cancer patients, these delays could extend much further with a figure of greater than 600 days given [21].

Attributing costs to our trials was also difficult. Despite the fact that NHS partner sites have ways to reclaim the costs of undertaking research, advice regarding how to interpret the cost allocation guidance (‘Attributing Revenue Costs of non-commercial research in the NHS (ARCO)’ [22]) was inconsistent. Warlow similarly highlighted that the regulations governing who pays for what in research in the NHS do not work [23]. Having to navigate through these governance processes has been met with significant bureaucracy, delay and costs [19,21,23-25].

To conduct medical research in the UK in the face of interminable delays and spiralling costs is decreasingly attractive to researchers, clinicians and funders. Professor Sir Michael Rawlins, in summing up the evidence of the Academy of Medical Sciences report reiterated this: “We have found unequivocal evidence that the health research in this country is being jeopardised by a regulatory and governance framework that has become unnecessarily complex and burdensome. Further, we received no evidence that this increased regulatory and governance burden has led to enhanced safeguards for participants in research” [21]. The report proposed a number of recommendations: to create a new Health Research Agency to rationalise the regulation and governance of all health research; to have a single system for ethics approvals; to improve the UK environment for clinical trials; to provide better access to patient data for approved research; and to embed a culture that values UK research [21].

We fully endorse the findings of the Academy of Medical Sciences report and while conscious that the issues are complex, on the basis of our experiences in these three trials, we recommend that the research governance systems could be improved in a number of ways. There are some populations where normal consent procedures are not possible. Research in emergency care or involving elderly patients are two such examples. The National Research Ethics Service should issue clear guidance to RECs on acceptable recruitment and consent procedures for these populations at risk, in order to avoid protracted discussions regarding the most appropriate procedures. Many NHS R&D departments still work independently and undertake their own processes of review, despite approvals already having been approved elsewhere. The NIHR should issue standard operating procedures for local NHS R&D departments to ensure practises are standardised. Whilst undertaking these trials we encountered difficulties regarding the appropriate attribution of costs, based on varying advice received. Even when attribution of costs had been agreed, local level agreement and sign-up to reimbursement was difficult. The guidance for attribution of costs for undertaking research within the NHS needs to be further clarified, with one body taking sole responsibility for all costs (research, excess treatment and service support costs) associated with undertaking research.

## Conclusions

Following the publication of the Research Governance Framework for Health and Social Care, medical researchers have been subjected to numerous additional regulatory and application processes. Although designed to safeguard the participants and researchers involved, introduction of these regulatory processes has often resulted in significant time delays and inflated research costs. Further streamlining and standardisation of these processes in needed to ensure that high-quality research can continue in the UK.

The competitive advantage of a publicly funded, open access health system for undertaking health services research and clinical trials has been outweighed in recent years by stifling bureaucratic structures and processes for governance of research in the UK. New guidance from the Academy of Medical Sciences and plans set out in this year’s budget for a Health Research Regulatory Agency to streamline the regulations on clinical trials, and so make them cheaper and easier to run [26], present another opportunity to tidy up structures and processes. As researchers, we await with interest the effects in practice on the time and cost of set up and delivery of publicly funded NHS based research in the UK.

## Competing interests

The authors declare that they have no competing interests.

## Authors’ contributions

HS and ITR conceived the idea for the manuscript, SS-B, AS and JGW contributed data for the review. HS and HH collated the trial data and drafted the report for submission to the Academy of Medical Sciences call for evidence. HS and HH drafted the manuscript. All authors read and edited the final manuscript.

## Pre-publication history

The pre-publication history for this paper can be accessed here:

http://www.biomedcentral.com/1471-2288/12/122/prepub
